# Surface modified cellulose scaffolds for tissue engineering

**DOI:** 10.1007/s10570-016-1111-y

**Published:** 2016-11-09

**Authors:** James C. Courtenay, Marcus A. Johns, Fernando Galembeck, Christoph Deneke, Evandro M. Lanzoni, Carlos A. Costa, Janet L. Scott, Ram I. Sharma

**Affiliations:** 1 Centre for Sustainable Chemical Technologies, University of Bath, Claverton Down, Bath, BA2 7AY UK; 2 Department of Chemical Engineering, University of Bath, Claverton Down, Bath, BA2 7AY UK; 3 National Nanotechnology Laboratory, Centre for National Research in Energy and Materials, Campinas, São Paulo Brazil; 4 Present Address: Department of Chemistry, University of Campinas, Campinas, Brazil; 5 Department of Chemistry, University of Bath, Claverton Down, Bath, BA2 7AY UK

**Keywords:** Bacterial cellulose, Surface modification, Cell adhesion, Tissue engineering scaffolds

## Abstract

We report the ability of cellulose to support cells without the use of matrix ligands on the surface of the material, thus creating a two-component system for tissue engineering of cells and materials. Sheets of bacterial cellulose, grown from a culture medium containing *Acetobacter* organism were chemically modified with glycidyltrimethylammonium chloride or by oxidation with sodium hypochlorite in the presence of sodium bromide and 2,2,6,6-tetramethylpipiridine 1-oxyl radical to introduce a positive, or negative, charge, respectively. This modification process did not degrade the mechanical properties of the bulk material, but grafting of a positively charged moiety to the cellulose surface (cationic cellulose) increased cell attachment by 70% compared to unmodified cellulose, while negatively charged, oxidised cellulose films (anionic cellulose), showed low levels of cell attachment comparable to those seen for unmodified cellulose. Only a minimal level of cationic surface derivitisation (ca 3% degree of substitution) was required for increased cell attachment and *no* mediating proteins were required. Cell adhesion studies exhibited the same trends as the attachment studies, while the mean cell area and aspect ratio was highest on the cationic surfaces. Overall, we demonstrated the utility of positively charged bacterial cellulose in tissue engineering in the absence of proteins for cell attachment.

**Electronic supplementary material:**

The online version of this article (doi:10.1007/s10570-016-1111-y) contains supplementary material, which is available to authorized users.

## Introduction

Damaged tissues and organs are a costly problem in healthcare, which, in some cases, cannot be addressed using traditional medical intervention (Song and Ott 2011). Tissue engineering approaches to rectify damaged tissue and organs are proving to be a viable alternative to transplantation, prosthetics, and surgical intervention. These approaches entail culturing cells on scaffolds that are placed into the injury site (Salgado et al. 2013). The scaffold serves as a support for the cells and provides a 3D framework for the cells to proliferate, produce extracellular matrix and generate tissue (Agrawal et al. 2014a, b, c). Scaffolds can be constructed from synthetic or natural biomaterials, but should be biocompatible, promote cell attachment and growth, and degrade over time (Hollister et al. 2002; Agrawal and Ray 2001).

Scaffolds derived from synthetic polymeric materials may offer advantages over natural biomaterials, such as reproducibility; their well-defined chemical composition can allow for precise control over mechanical properties and degradation rates (Okamoto and John 2013). However, synthetic biomaterials suffer from a major disadvantage as they often lack sites for cell adhesion; therefore, many need to be modified to introduce cell attachment cues, such as matrix ligands, for adhesion (O’Brien 2011). The addition of ligands or peptides may be achieved by passive adsorption (simplest method) (Cutler and García 2003), or more complex routes such as incorporation into the polymer backbone (Schmedlen et al. 2002), at the ends of the polymer chains (Hersel et al. 2003), or functionalised on the material surfaces (Wan et al. 2004). In general, these approaches involve complex chemistries, or costly crosslinking reagents that are unstable after a short period of time, adding cost and complexity to production. Furthermore, some are poorly biocompatible and may cause inflammation or immune responses when implanted or upon degradation in vivo (the degradation products can also be deleterious) (Willerth and Sakiyama-Elbert 2008). Natural scaffolds are often biocompatible with the implant tissue (Peloso et al. 2015; Abouna 2008), but the origin of the scaffold material can lead to complications: many are from animal sources, which may offend some religious sensitivities and personal beliefs. In addition, concerns may arise pertaining to transmission of pathogens, such as including prions.

Common synthetic polymers used in tissue engineering include poly(lactic-co-glycolic acid), PLGA, and poly(ethylene glycol), PEG. PLGA is a biocompatible, polyester copolymer of lactic and glycolic acids, which degrades in vivo. Due to its tuneable mechanical properties, it has been used to prepare biodegradable scaffolds for a range of applications including: bone grafts (Agrawal et al. 2014a, b, c); to generate adipose tissue for reconstructive surgery (Neubauer et al. 2005); and spun into fibres for seeding cells (Teng et al. 2002). However, when PLGA degrades in vivo, the acidic metabolites can have a detrimental effect on the local pH of the extracellular matrix (ECM), which can cause inflammation and an immune response, or even cell and tissue necrosis (Willerth and Sakiyama-Elbert 2008; Liu et al. 2006). Hydrogels prepared from PEG are able to resist protein adsorption due to the non-ionic hydrophilic nature of the polymer (Knop et al. 2010) and have been used to engineer a wide range of tissue from bone (Luu et al. 2003) and cartilage (Bryant and Anseth 2003) to nerve tissue (Cai and Kim 2010). However, like PLGA, PEG scaffolds often need to be functionalised with matrix ligands or peptides to facilitate cell attachment.

In spite of the potential variability in composition of natural biomaterials, protein derived scaffold materials, such as collagen, fibrin and glycosaminoglycan (Patterson et al. 2010) often possess the chemical structures that can mimic native tissue, thereby aiding biocompatibility (Agrawal et al. 2014a, b, c). For example, collagen type I (a key component of the ECM), can be reconstructed into a fibrillar matrix beneficial for cell attachment and has been formed into hydrogel sponges used for bone and tissue repair (Glowacki and Mizuno 2008). Decellularised tissue and organs have also been used in a variety of tissue engineering applications (Song and Ott 2011; Provencher et al. 2007). However, the risk of immunogenicity and disease transmission can remain after treatment. Cells are removed from donor tissue to prevent recognition by the host, avoiding an inflammatory response, or an immune-mediated rejection of the tissue (Gilbert et al. 2006). The remaining tissue is a complete ECM, which can closely match the damaged tissue (Crapo et al. 2011). However, as the source of material is a deceased donor (for most organs), this is not a sustainable supply. Aging of donor tissue leading to biochemical and mechanical changes (Blevins et al. 1991) and variation in properties with origin, as well as alteration in the decellularisation process, may also render this scaffold type less useful (Gilbert et al. 2006).

There is a need for a new biomaterial with suitable properties for tissue engineering, derived from a sustainable source, and which requires minimal processing to achieve cell viability for industrial application. Cellulose has the potential to fulfil these requirements, as it is: the most abundant biopolymer on earth, found in plant cell walls and produced by certain bacteria such as *Acetobacter* (Eyley and Thielemans 2014); chemically homogeneous, being constructed from anhydroglucose units connected by β-1,4 glycosidic bonds (Agrawal et al. 2014a, b, c); biocompatible (Klemm et al. 2005); has tuneable tensile strength (Syverud et al. 2015); and can be readily functionalised as it bears three accessible OH groups per repeat unit, which are available for a vast range of modifications (Isogai et al. 2011; Agrawal et al. 2014a, b, c; Peng et al. 2011; Ma and Ramakrishna 2008).

Scaffolds prepared from cellulose have been considered previously for tissue engineering. However, as cellulose is a hydrophilic material with low non-specific protein adsorption (which is why mammalian cells do not readily adsorb to cellulose surfaces) (Wu et al. 2003; Zou et al. 2001; Pelton 2009; Brash and Ten Hove 1993), these scaffolds required the addition of matrix ligands, to facilitate cell attachment to their surfaces (Singh et al. 2013; Modulevsky et al. 2014; Torres-Rendon et al. 2015; Feldmann et al. 2015). Watanabe et al. (1993) demonstrated that, by introducing an ionic charge to cellulose membranes, collagen could be adsorbed to the membrane surface to promote cellular adhesion.

Here we investigate whether the introduction of a surface charge on cellulose films, through simple chemical derivitisation, will increase cell attachment, without the use of matrix ligands. To introduce a positive charge the epoxide, glycidyltrimethylammonium chloride (GTMAC), was grafted onto cellulose through the nucleophilic addition to the alkali-activated cellulose hydroxyl groups (Zaman et al. 2012). The radical 2,2,6,6-tetramethylpipiridine 1-oxyl (TEMPO) was used to mediate the oxidation of the primary alcohols to introduce a negative charge (Isogai et al. 2011). This methodology allowed for a novel application using cellulose films that were surface modified by derivitisation.

## Materials and methods

To produce bacterial cellulose the *Acetobacter* organism was sourced from Happy Kombucha (UK). Glucose, yeast extract, peptone, anhydrous disodium phosphate and citric acid monohydride were all purchased from Sigma-Aldrich (UK) and used as received.

For surface modifications, sodium hydroxide pellets (≥98%), glycidyltrimethylammonium chloride (GTMAC) (≥90%), 0.1 M AgNO_3_ aqueous solution (≥95%), 2,2,6,6-tetramethylpipiridine 1-oxyl radical (TEMPO) powder, NaBr powder, NaOCl 5.00 vol% solution, HCl (reagent grade) were purchased from Sigma-Aldrich. Aqueous solutions of AgNO_3_, NaOH and HCl were made up to the required concentrations with deionised (DI) water.

For cell investigations Dulbecco’s Modified Eagle Medium (DMEM) (GlutaMAX™), non-essential amino acids (NEAA), sodium pyruvate (NaPyr), trypsin (0.05%) and trypan blue (0.4%) were purchased from Gibco^®^ and stored at 4 °C. Foetal bovine serum (FBS) (non-USA origin), MG-63 cells, RGD-peptide and formaldehyde (37% in 10–15% methanol H_2_O solution) were purchased from Sigma-Aldrich^®^. Phosphate buffer solution (PBS) was purchased from HyClone^®^ (0.1 μm sterile filtered), 6-diamidino-2-phenylindole (DAPI), phalloidin-FITC and penicillin streptomycin (PenStrep) from Life Technologies. Norland optical adhesive 63 was purchased from Norland Products. All materials were used as received.

Polystyrene latex beads (0.3 µm) were purchased from Sigma-Aldrich and used as tracer particles for zeta-potential measurements.

### Preparation of bacterial cellulose films

Sheets of bacterial cellulose (30 cm × 50 cm) were produced under culture conditions following (Dufresne 2012).

The cellulose sheets were sterilised (and bleached) by treatment for 2 h in 2 L of 5% sodium hypochlorite in DI water, followed by thorough washing in 2 L aliquots of DI water. The cleaned sheets were stored in 2 L of 20% methanol in DI water solution to prevent fungal growth. Cellulose sheets were cut into 5 cm^2^ squares, placed on glass petri dishes and dried under vacuum at 50 °C for 24 h (yielding <2% of the original wet mass). The remaining moisture content was determined by thermogravimetric analysis and dried cellulose sheets were stored in sealed polyethylene bags.

### Surface modification by derivitisation and oxidation

#### Cationic-cellulose

Following the semi dry procedure described by Zaman et al. (2012). 5 wt% NaOH (relative to corrected film mass) dissolved in 5 mL of DI water, was added to the cellulose films contained in polyethylene bags. Accurately weighed GTMAC (0.60–1.05 g) in molar ratios of 0.5–3.0, relative to anhydroglucose units (AGUs) of the weighed cellulose, was added drop wise and the sample kneaded to achieve homogenisation, prior to reaction at 65 °C (water bath) for 75 min. Modified cellulose films were washed thoroughly in DI water before being dried under vacuum at 50 °C for 24 h. These GTMAC modified films will be referred to as “cationic–cellulose” in this paper.

The degree of substitution was determined by conductometric titration of chloride ions (trimethylammonium chloride groups) with AgNO_3(aq)_. Squares of film (2 × 2 cm, 10–50 mg) were accurately weighed and immersed in 20 mL of DI water for 5 min. Titrant (0.837 mM AgNO_3_) was added in 0.50 mL aliquots and the conductivity was monitored using a SevenMulti Mettler Toledo conductivity probe. The degree of substitution is calculated using Eq. 1:

1 $$\rm{Degree\;of\;substitution}\;\% = \left[ {\frac{{162.15 - \left( {C \times V} \right) }}{{w - \left( {151.63 \times C \times V} \right)}}} \right]100$$

where C is the concentration of AgNO_3_ solution (M), V is the volume of AgNO_3_ solution (in dm^3^), and w is the weight of the dried cationic cellulose sample (g), 162.15 is the M_w_ of the AGU and 151.63 is the difference in M_w_ between the AGU and cationised AGU with trimethylammonium chloride group. Triplicate samples were analysed for each material and an average reported.

#### Anionic-cellulose

TEMPO (0.016 g, 0.1 mmol) and NaBr (0.1 g, 1.0 mmol) was added to 200 mL DI water in an ice bath. Accurately weighed dry bacterial cellulose films (1–2 g) were submerged in the solution for 10 min. The pH of 5 vol% NaOCl solution was adjusted to 10 with 0.1 M HCl and a quantity equivalent to 0.05–0.30 mol equivalents, relative to AGU, added drop wise to the film containing solution, under constant stirring, the pH was maintained at 10 by drop wise addition of 0.5 M NaOH _(aq)_ when required. Ethanol (10 mL) was added to quench the reaction and the films were washed thoroughly in DI water and dried. These modified films will be referred to as “anionic-cellulose” in this paper.

The carboxylate content of the anionic-cellulose films was determined by conductometric titration; 50 mg anionic cellulose samples (accurately weighed) were immersed in 15 mL of 10.00 mM HCl standard solution for 10 min. Titrant (10.00 mM NaOH) of was added in 0.50 mL aliquots and conductivity monitored using a SevenMulti Mettler Toledo conductivity probe. The degree of oxidation is calculated using Eq. 2:

2 $$\rm{Degree\;of\;oxidation}\;\% = \left[ {\frac{{162.15 \times C \times \left( {V_{2} - V_{1} } \right)}}{{w - \left( {35.97 \times C \times \left( {V_{2} - V_{1} } \right)} \right)}}} \right] 100$$

where C is the concentration of NaOH solution (M), V is the volume of NaOH solution (in dm^3^), w is the weight of the dried anionic cellulose sample (g), 162.15 is the M_w_ of the AGU and 35.97 is the difference in M_w_ of AGU and sodium salt of the glucoronic acid group (Zaman et al. 2012). Triplicate samples were analysed for each material and an average reported.

#### Characterisation

^1^H–^13^C CP/MAS NMR was performed on unmodified, cationic (DS = 3.0 ± 0.0%) and anionic (DO = 7.6 ± 1.0%) cellulose powders (freeze dried). Spectra were acquired at 25 °C, an MAS rate of 10 kHz and a contact time of 2000 µs. FTIR spectra for unmodified, cationic (DS = 3.0 ± 0.0%) and anionic (DO = 7.6 ± 1.0%) cellulose powders were obtained on a Perkin Elmer Spectrum 100 with a universal ATR sampling accessory; 10 scans were acquired in the range 4000–600 cm^−1^.

The presence of quaternary ammonium, or carboxylic acid, functional groups was confirmed by both FTIR and solid-state ^13^C NMR measurements. FTIR: prominent bands at 1440 and 1483 cm^−3^ were attributed to the CH_2_ bending mode and methyl groups of the cationic cellulose substituents in accordance with data published by Zaman et al. (2012) ^13^C solid-state NMR: signals between 66 and 105 ppm referred to the anhydroglucose, while a signal at 175 ppm appeared upon oxidation, due to the carboxylic acid group (Saito et al. 2005) and a signal at 56 ppm due to the methyl groups on the quaternary ammonium was detected in the cationic cellulose sample (Chaker and Boufi 2015) (Figs. S1, S2, Supplementary information).

### Scaffold characterisation

#### Zeta potential measurements

The surface ζ-potentials of unmodified, cationic and anionic bacterial cellulose films were measured at 25 °C using a Malvern Zetasizer Surface ζ-Potential Cell. Films were cut into 4 × 4 mm pieces, adhered to the sample plate and placed between the electrodes of the measurement cell. The position of the sample plate was aligned to the laser height. An aqueous suspension of 0.3 μm polystyrene latex tracer particles was prepared and 1.50 mL added to a 3 mL cuvette. The measurement cell was inserted into the cuvette ensuring no air bubble was trapped underneath the film. The application of an electric field via the electrodes initiated electrophoresis of the particles and electro-osmosis close to the surface.

The measured electrophoretic mobility of the tracer particles will vary as a function of distance from the sample surface. By plotting the reported mobility (ζ-potential) as a function of displacement from the surface, the relationship can be extrapolated back to the intercept (zero displacement). Therefore, the surface ζ-potential can be defined by Eq. 3.

3 $$\zeta \;\rm{film}\;\rm{surface} = - \rm{intercept} + \zeta \rm{particle}$$

Triplicate samples were analysed for each material, the measurement repeated fifteen times per sample, and an average reported.

#### Scanning probe microscopy

Topography and capacitance gradient (*dC*/*dz*) images of unmodified, cationic and anionic cellulose films were obtained using a Park NX-10 Atomic Force Microscope (Gouveia and Galembeck 2009; Ferreira et al. 2015). PPP-EFM probes (NanoWorld) with spring constant of 2.8 N/m and resonance frequency within 75 kHz were used for measurements. Topography and electrical images were acquired in air by single pass scanning at room temperature and humidity between 74.5 and 75.5%. Topography was measured using the intermittent contact mode setup, slightly below the frequency of resonance. Kelvin force and capacitance coupling measurements were conducted in parallel by applying an electric AC signal at 17 kHz to the metal-coated cantilever. The electrical potential of the sample is deduced by the DC potential applied to the cantilever to nullify the AC signal at 17 kHz. Furthermore, the second harmonic of the AC signal (34 kHz), which is shown to be proportional to the capacitance gradient (*dC*/*dz*), or capacitance coupling, of the tip to the sample, was monitored. Analysis and processing of the AFM images were carried out with Gwyddion (Necas and Klapetek 2012). The capacitance coupling signal distribution was calculated using the 1D height analysis function of the programme.

#### Mechanical testing

The Young’s modulus and tensile strength of the scaffolds were determined using an Instron 3343 electromechanical test machine. The samples used were unmodified, cationic (3.6 ± 0.3% degree of substitution) and anionic (6.7 ± 0.6% degree of oxidation) cellulose films. The films were cut into strips ≥1.50 cm in length by 0.30 or 0.50 cm width and the thickness recorded with a steel digital vernier micrometer calliper. The film strips were glued onto card mounts and the adhesive was allowed to set, which prevented damage to the films prior to characterisation. The mounts were gripped between the vices and a 1000 N cell was used to deliver strain to the films until deformation, or failure. Five samples were tested for each film and an average reported.

### Cell adhesion

#### Preparation of scaffolds

Films (unmodified or modified) were cut to a size that fit into a well plate and washed with DI water. The films were placed into a well plate (Costar^®^, Tissue culture-treated well plates, which were used as the control substrate throughout) and sterilised in a Hoefer UVC 500 cross linker for 15 min. After this time the films were turned over with sterilised tweezers and the sterilised side adhered to the well plate with a single drop of Norland optical adhesive 63. The well plate and contents were resterilised (15 min irradiation), PBS (1 mL) was added to each well and the plate stored at 4 °C.

Under sterile conditions, the PBS was removed from the films and 300 μL of DMEM medium, either alone, or with pure FBS or RGD solution (10 μg/mL) as appropriate, added to the wells and left to hydrate for 24 h at 4 °C prior to cell attachment studies.

#### Cell attachment

Once the films had been hydrated with the relevant medium for 24 h, the medium was removed under sterile conditions and the scaffolds were seeded at a seeding density of 20,000 cells/cm^2^ from a suspension of MG-63 cell culture of a known concentration. An empty well plate was seeded as a control. Growth medium (500 μL) was added to each well, which was then sealed and placed in a CO_2_ incubator for 1 h. The samples were tested in triplicate.

#### Cell fixation and DAPI staining

The medium (and unattached cells) was aspirated from the wells and scaffolds and cells fixed: 2× wash with 1 mL PBS; treatment with 1–2 mL of 3.7% formalin (1 mL formaldehyde solution diluted to 9 mL with PBS) for 15 min at RT; followed by 2× wash with 1 mL PBS; then stained under low light level conditions: 2× wash with 1 mL PBS; 15 min staining with DAPI solution (300 μL of DAPI in PBS, 1 μL in 50 mL); 2× wash with 1 mL PBS. A final 1 mL PBS was added to the scaffold, the plate wrapped in aluminium foil and stored at 4 °C.

#### Analysis of cell attachment

Under low light levels, the films were removed from the well plate and placed cell side down on glass microscope slides for viewing with an EVOS optical microscope using blue light. Six independent images of the film surface were obtained using a 10× objective and cells counted using the “cell count” function in ImageJ, normalised to the area of field of view. The average count from the six images was used to determine the percentage cell attachment, using Eq. 4.

4 $$\% \;{\text{cell}}\;{\text{attachment}} = \frac{{{\text{No}}.\;{\text{of}}\;{\text{cells}}\;{\text{on}}\;{\text{scaffold}}}}{{{\text{Seeding}}\;{\text{density}}}} \times 100$$

#### Cell adhesion

Scaffolds were prepared as described for cell attachment experiments. After 1 h incubation the seeded scaffolds were centrifuged at 200 rpm (8*g*) for 10 min, following which the cells were fixed and stained with DAPI. Attachment was determined as described above.

#### Cell morphology

PBS was removed from hydrated films, which were seeded at 2500 cells/cm^2^ in serum free medium and incubated for 1 h. Following which, the medium was removed and replaced with FBS containing medium (performed gently with a pipette, ensuring the attached cells were not disturbed during the process). Cells were fixed, permeabilised, and stained with 200 μL of dilute phalloidin-FITC solution (100 μL in 10 mL PBS) for 40 min at RT, followed by washing with two 1 mL aliquots of PBS solution. A final 1 mL PBS was added to the scaffold, the plate wrapped in aluminium foil and stored at 4 °C. Cells were visualised as above, with the exception that phalloidin-FITC stains F-actin in the cytoskeleton, thus providing a fluorescent image of the cell. The degree of cell spreading was inferred from measurements of area and aspect ratio. Six independent images of the film surface were taken with a 10× objective and the average value reported.

Images were analysed using ImageJ following the method described by Fardin et al. (2010). Projected cell area and aspect ratio were used in combination to quantify changes in cell morphology over 24 h.

### Statistical analysis

Triplicate data were analysed using IBM SPSS Statistics Data Editor. A one-way analysis of variance (ANOVA) was used to determine whether there were any statistical differences between the means of two or more independent measurements, assuming equal variance. The differences were considered significant at the level of *p* < 0.001(***), *p* < 0.01(**) and *p* < 0.05(*).

## Results

### Surface modification and characterisation

**Scheme 1:**
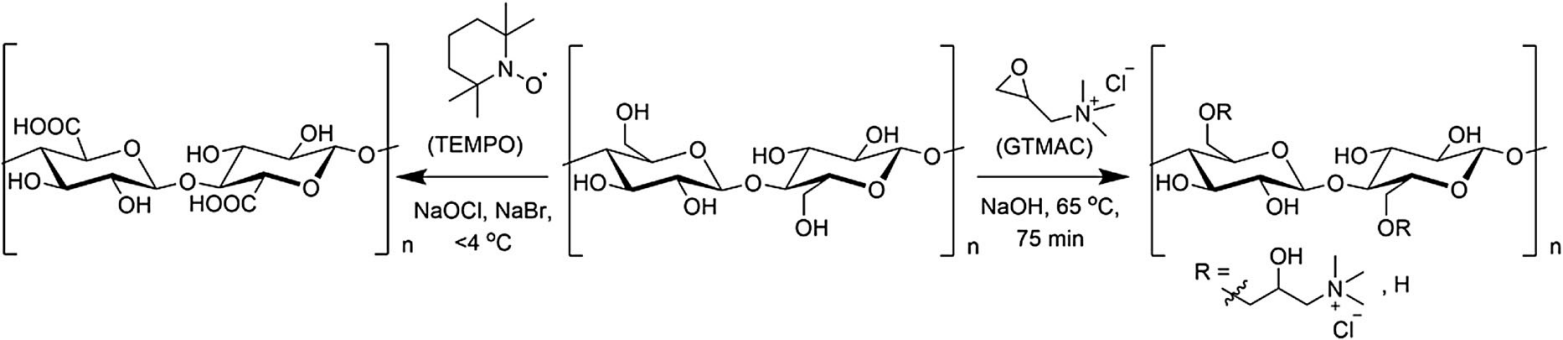
Cationisation of cellulose films with GTMAC following activation of cellulose alcohol functionality by treatment with NaOH (*right*). Oxidation of C6 1° alcohol groups to C6 carboxylate groups pH 10–11 (*left*). In both cases the reaction is primarily with primary OH groups accessible on the film surface and the degree of substitution, or oxidation, is controlled by modulating the quantity of reagent (GTMAC) or oxidant (NaOCl) added

Cellulose surfaces were rendered positively charged by nucleophilic substitution of alkali activated 1° alcohol groups with an epoxide bearing a quaternary ammonium group (GTMAC), or negatively charged by controlled oxidation using the well-studied TEMPO/NaOCl/NaBr system (Scheme 1).

**Fig. 1:**
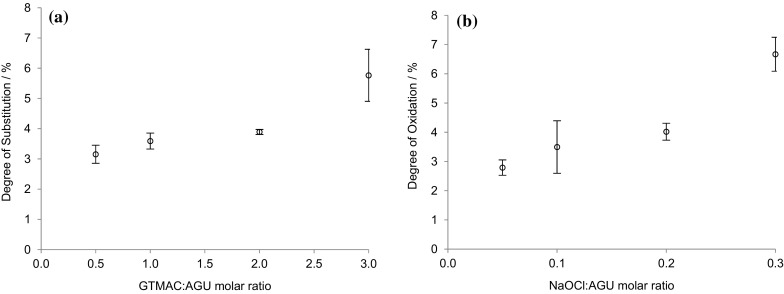
**a** DS and **b** DO per anhydrous glucose repeat unit for the modified bacterial cellulose films determined by conductometric titration. The average of three values was reported with the standard deviation shown as *error bars*

The degree of surface modification was controlled by modulating the quantity of reagent (GTMAC), or oxidant (NaOCl), and the degree of substitution (DS), or oxidation (DO), of cellulose films assessed using conductometric titration (Fig. S3, Supplementary information). While, in both cases, the extent of introduction of charged groups increased with increased molar ratio of reagent, or oxidant, to AGU (Fig. 1), it is clear that the oxidation reaction is significantly more efficient than the derivatisation. The former yielded oxidation levels between 3 and 4% at an NaOCl:AGU ratio <0.1, while a ratio of 3:1 GTMAC:AGU was required to achieve a similar level of substitution.

GTMAC was successfully grafted onto the surface hydroxyl groups of α-cellulose producing cationic cellulose. The cellulose films were functionalised with a DS value between 3.2 and 5.8% and a DO of 2.7–6.7%. This showed that the degree of modification on the surface could be controlled.

### Mechanical properties

Bulk mechanical properties of the unmodified and modified bacterial cellulose films were compared to discern if modification of surface chemistry was likely to compromise the integrity of the films. It is known that oxidation of fibrous cellulose leads to some loss of material (presumably by dissolution) and individualisation of fibrils, thus a film of relatively high DO was selected for comparison (Jin et al. 2014).

**Fig. 2:**
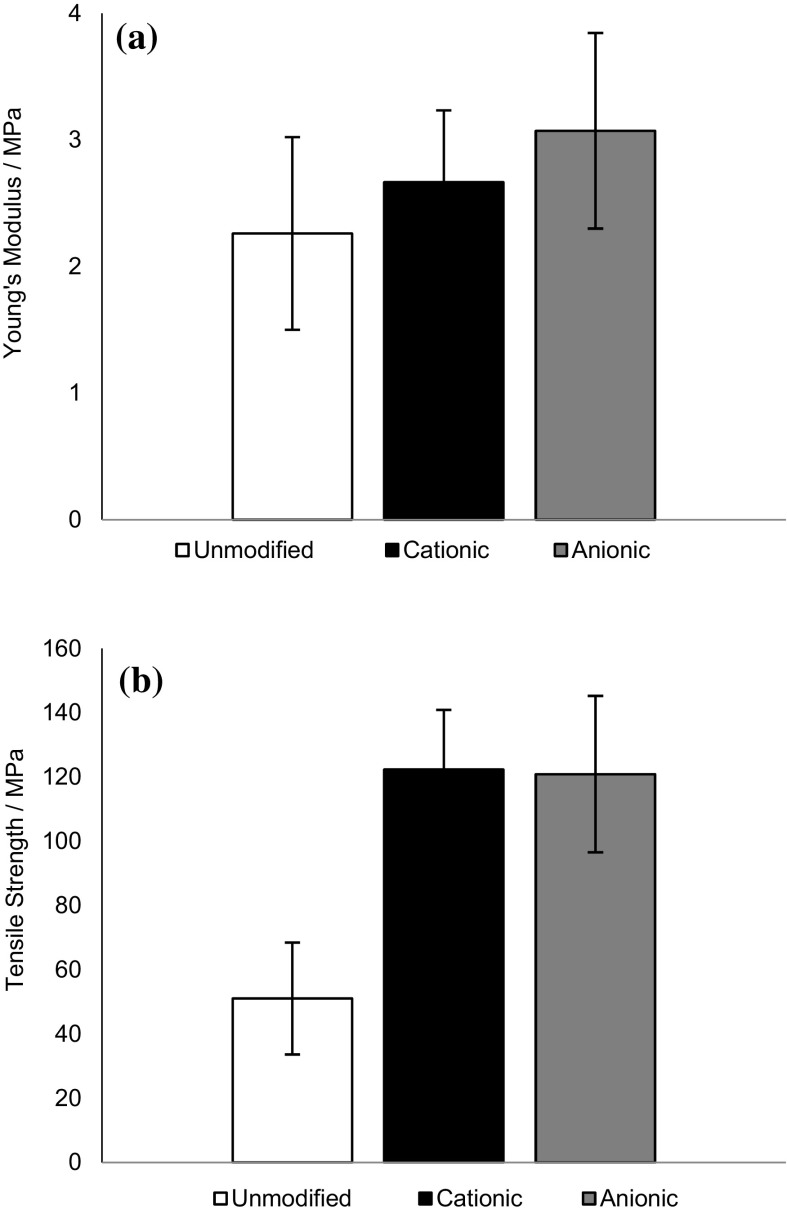
**a** Young’s modulus and **b** tensile strength of unmodified, cationic (3.6 ± 0.3% DS) and anionic (6.7 ± 0.6% DO) cellulose films, n = 5. The average of five values was reported with the standard deviation shown as *error bars*

The Young’s modulus for the unmodified cellulose films was 2 ± 0.8 MPa [comparable with previously reported value of 1.6 MPa (Zaborowska et al. 2010)] and did not change significantly upon modification (Fig. 2a). Tensile strength appeared to increase significantly upon modification (Fig. 2b), providing confidence that, even at the highest DS and DO values tested, film strength was not compromised. It was postulated that the strengthening of the modified films was due to increased density of packed fibrils within the films, as the modified films exhibited thickness of only 60–80% that of unmodified films, reflecting previous reports that films made from modified cellulose fibrils possess higher tensile strengths than native cellulose (Tanaka et al. 2016).

### Surface ζ-potential and capacitance

**Fig. 3:**
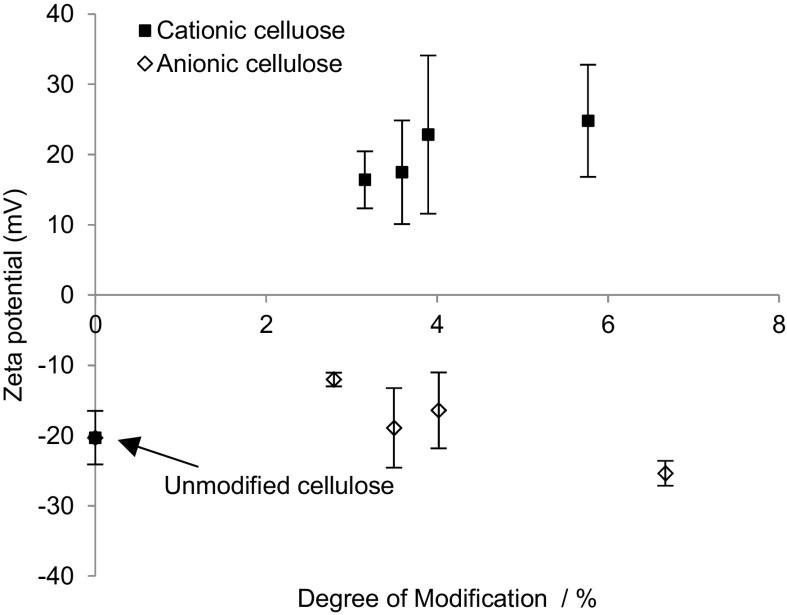
The ζ-potential measurements on modified cellulose films confirmed that the cationic surfaces were indeed positive and anionic negative, n = 3. The average of three values was reported with the standard error shown as *error bars*

To discern the effect of modification on surface charge, the surface ζ-potential was measured for each of the modified films (Fig. 3)

.

The measured ζ-potential for unmodified cellulose films was −20 ± 4 mV, indicating that, prior to alteration of surface chemistry, the cellulose films bear some surface functionality that imparts anionic character to the materials (in agreement with previous reports, where a value of −8 mV was reported (Lee et al. 2011)). When derivatised with GTMAC, the ζ-potential increased to 25 ± 9 mV due to the introduction of the positively charged trimethylammonium groups. Oxidised cellulose exhibited a negative value, as expected, but this was not significantly different from underivatised cellulose.

**Fig. 4:**
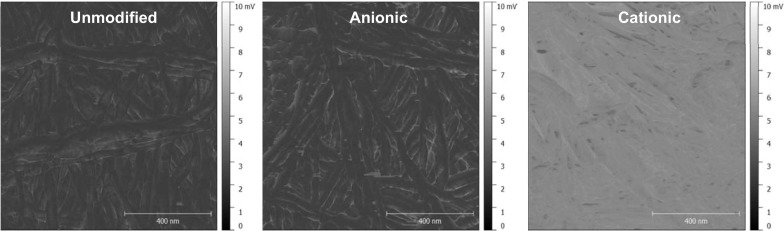
Capacitance gradient (*dC*/*dz*) images of unmodified, cationic and anionic cellulose films were obtained over a 1 µm^2^ sample. The capacitance coupling of the tip to the sample was measured and determined by the capacitance of the probed sample volume. The scale is in mV as a signal is generated that is linearly proportional to *dC*/*dz*. The *black*/*white* scale indicates the magnitude of *dC*/*dz* signal of the sample, whereby *black* = 0 and *white* = 10 mV. The cationic cellulose surface is a *lighter shade* which reflects a higher capacitive coupling *dC*/*dz*

**Fig. 5:**
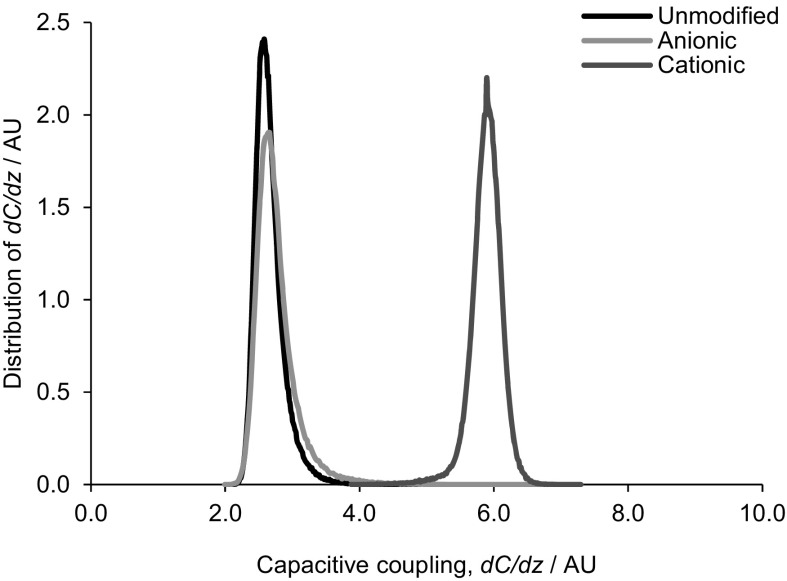
The capacitive coupling distribution between the tip and surface was generated by a 1D statistical analysis of images depicted in Fig. 4, for the unmodified, anionic and cationic cellulose films. Capacitance coupling was measured across a 10 µm^2^ sample surface area. The peak at 5.9 AU indicates that the cationic cellulose has a higher capacitive coupling, *dC*/*dz*

To compare films, both with respect to surface charge and charge distribution (homogeneity), electric force microscopy was employed to characterise changes in capacitive coupling (proportionally to the mobility of charge) of the tip to the film surface, *dC*/*dz* (Fig. 4). Clearly, unmodified and anionic cellulose surfaces exhibit similar capacitance coupling (mirroring the ζ-potential measurements), while the cationic material exhibits a significantly greater capacitive coupling, *dC*/*dz*, across the entire sample. This is reflected in Fig. 5, showing distribution of capacitive coupling over a larger area: both unmodified and oxidised surfaces exhibit similar surface capacitance coupling values of 2.6 arbitrary units (AU), while that of the cationised surface is 5.9 AU.

**Fig. 6:**
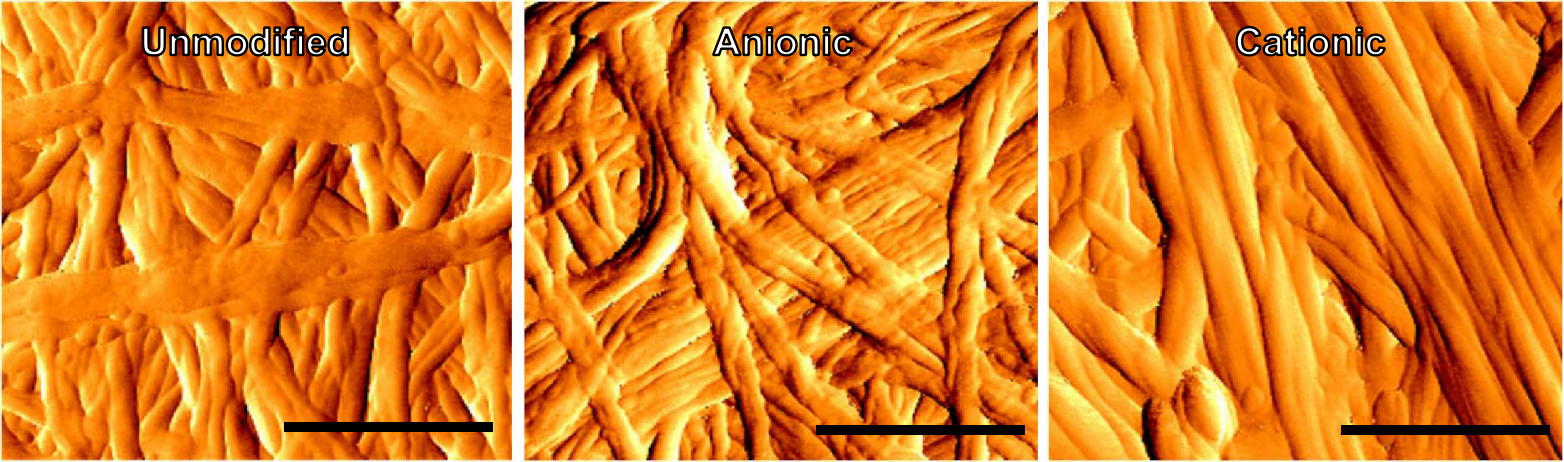
Tip amplitude image (error image) of the topography obtained of the surface over a 1 µm^2^ sample for unmodified, anionic and cationic cellulose films. The fibril network does not appear to have been degraded by the surface modification. *Scale bar* is 400 nm in length

The surface topography of the samples is reflected in the tip amplitude image of each film surface (Fig. 6) and only very minor differences noted. Unmodified films show the typical overlapping fibrillar structure of bacterial cellulose and this is reflected in both modified films; there is no significant change in the fibril dimensions.

### Cell attachment

**Fig. 7:**
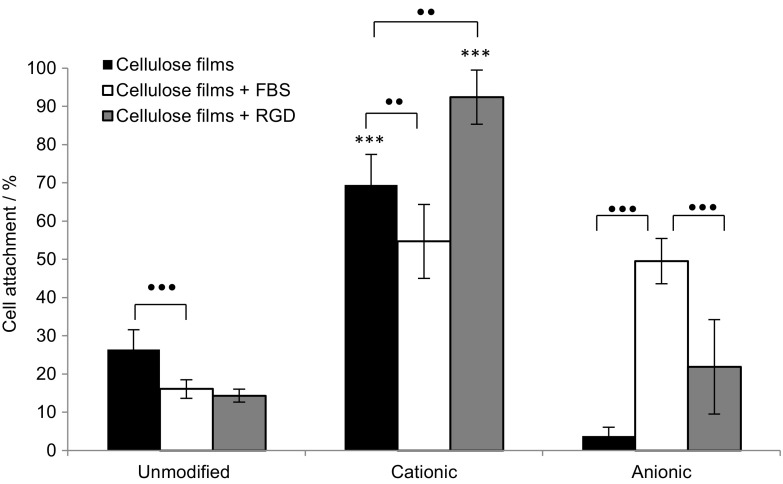
MG-63 cell attachment on cellulose films after 1 h incubation at 37 °C under 5% CO_2_, n = 3 and *error bars* show standard error. Films were immersed for 24 h prior to seeding in DMEM medium alone or DMEM medium containing FBS or RGD as appropriate. Significant cell attachment on cationic cellulose films was achieved without the need for FBS or RGD growth factors. Values significantly different from unmodified cellulose films were indicated by the confidence values *p < 0.05, **p < 0.01 and ***p < 0.001. To indicate significant differences between two values the symbol *black dot* was used to refer to the *p* value

Human osteoblast cancer cells, MG-63, were selected for their fibroblast phenotype and cell adhesion was tested in both the presence and absence of FBS and RGD to discern whether cell attachment could proceed without the need for added growth factors, or matrix ligands. A two component scaffold system reduces the cost of processing scaffolds and mitigates the implications of using animal derived ligands. After 1 h there was significantly greater cell attachment on the positively charged surfaces of cationic cellulose compared to the unmodified and anionic surfaces (Fig. 7). This difference was clearest in the *absence* of any added proteins and this is the first instance that direct cell attachment has been reported for modified cellulose scaffolds without mediation of FBS or RGD.

**Fig. 8:**
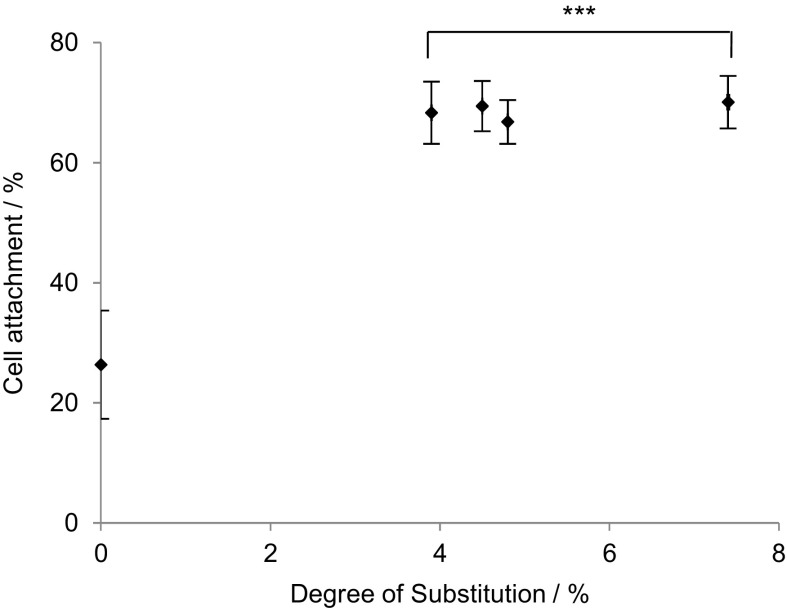
Influence of degree of substitution on MG-63 cell attachment on cationic cellulose films after 1 h incubation at 37 °C in 5% CO_2_, n = 3 and *error bars* show standard error. Films were immersed for 24 h in DMEM medium, prior to seeding. Only a minimal level of modification with GTMAC was required for significant enhancement of cell attachment on cationic cellulose surface versus unmodified cellulose. Values significantly different from unmodified cellulose films were indicated by the confidence values *p < 0.05, **p < 0.01 and ***p < 0.001

In the absence of mediating proteins, FBS and RGD, cell attachment to anionic cellulose was negligible, but some adhesion was recovered when scaffolds were pre-incubated with FBS, suggesting that surface charge is not the only important factor and surface chemistry may play a role in cell adhesion. Remarkably, the degree of substitution did not appear to have a significant affect with similar levels of cell attachment detected for all cationic cellulose films, regardless of DS (Fig. 8).

**Fig. 9:**
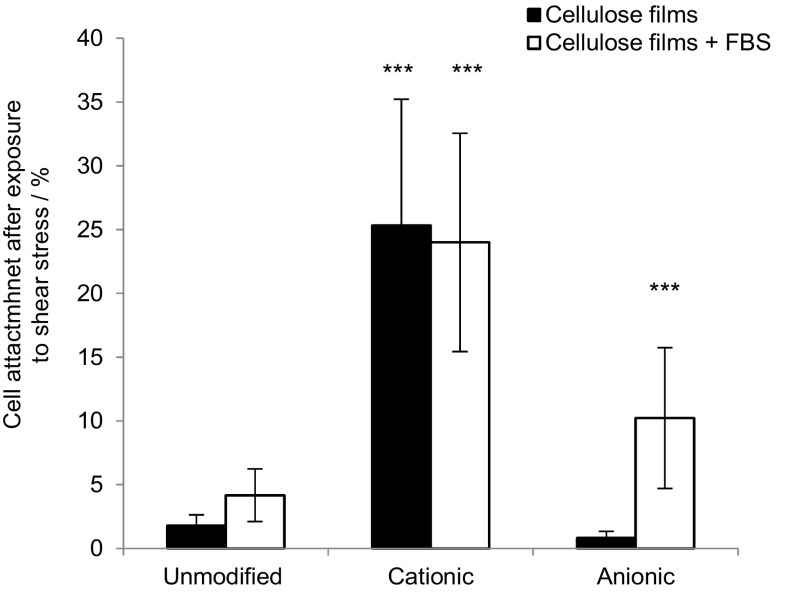
The percent of MG-63 cells attached on cellulose films after exposure to shear stress (centrifugation at 8*g*), n = 3 and *error bars* show standard error. Films were seeded in DMEM medium alone, or DMEM medium containing FBS. Values significantly different from unmodified cellulose films were indicated by the confidence values *p < 0.05, **p < 0.01 and ***p < 0.001

Cell adhesion strength was assessed by counting the percentage of cells remaining after centrifugation with and without FBS in the media (Fig. 9).

**Fig. 10:**
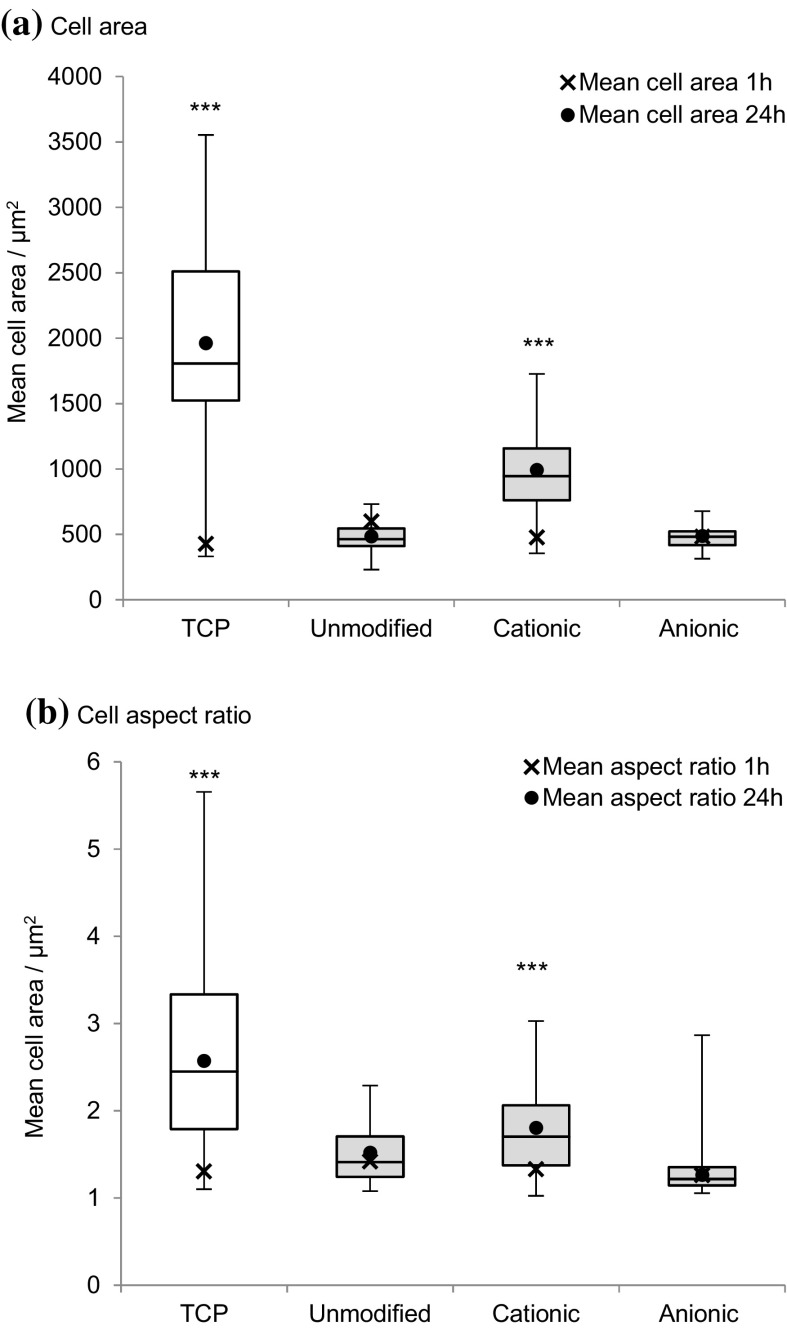
Change in MG-63 cell morphology; **a** cell area and **b** aspect ratio on cationic, anionic and unmodified bacterial cellulose scaffolds after 1 and 24 h incubation at 37 °C in 5% CO_2_, n = 17–53 cells measured and *error bars* show standard error. Cell images were analysed using ImageJ to calculate the average cell aspect ratio and area. The control was tissue culture plastic (TCP). Values significantly different from unmodified cellulose films were indicated by the confidence values *p < 0.05, **p < 0.01 and ***p < 0.001

To determine the cells' response to the substrate, the degree of cell spreading and morphology was characterised by the change in projected cell area and aspect ratio after 24 h incubation (Fig. 10). An increase in cell aspect ratio, through elongation, and cell area was noted on cationic cellulose, but minimal spreading was observed on the native and anionic cellulose scaffolds.

## Discussion

This is the first report of modulation of cell attachment on cellulose scaffolds induced by simple changes in surface chemistry of the cellulose scaffolds *without* mediation by added proteins. Cellulose in its natural form only permits minimal cell attachment, but when modified to have a positive charge, cell attachment increases to levels comparable to tissue culture plastic. Thus, we have established a minimally processed material for tissue engineering. The oxidation and derivatisation reactions employed are well known and thus easy to implement, offering opportunities to enhance, or indeed reduce, cell attachment simply by very minor alterations to (largely) the primary C6 hydroxyl groups exposed on the surface of cellulose scaffolds. Measurement of Young’s modulus and tensile strength suggest that these chemical modifications do not compromise the mechanical strength, or integrity, of the cellulose scaffold material and analysis by electrostatic force microscopy reveals that alteration of surface charge is reasonably homogeneous across the surface and that no significant changes in fibrillar morphology result. Together, these results suggest that oxidation, or derivatisation with GTMAC, at the low levels used here, result in modification of surface, rather than bulk, chemistry of the materials. While demonstrated here for bacterial cellulose, the chemistry of cellulose (a linear homopolymer of glucose with β 1–4 glycosidic linkages) is invariable between cellulose sources and this methodology would be expected to be extendable to a wide range of cellulose scaffolds.

Importantly, measurement of cell attachment values indicates that pre-treatment of the scaffolds ligands, in this case FBS (a protein serum supplement), prior to cell seeding, was not necessary for cell attachment to occur on the cationic cellulose scaffolds. (While attachment did occur in the presence of FBS, the results were somewhat more variable and no significant *improvement* in attachment was noted.) In a three-component system (cell, biomolecule, materials), containing FBS, matrix ligands will be dominant in mediating cell attachment, as their presence facilitates integrin binding and focal adhesion formation. FBS contains a cocktail of growth factors and proteins that will adsorb both to cationic and anionic cellulose surfaces. The influence of RGD, a simple peptide often used to enhance cell attachment, was also minimal. There are few *direct* studies of the influence of surface charge on cell attachment in the absence of, or without pre-treatment with, matrix ligands (Hamdan et al. 2006; Fotia et al. 2013). Thus, for the first time, we demonstrate that simply modifying the surface charge of a cellulose scaffold, by derivatisation using chemistry developed for the cloth dying industry, promotes attachment of cells (70% increase over unmodified cellulose scaffolds). This is significant as it reduces the cost of processing and preparing scaffolds and the implications of using animal derived proteins or synthetic peptides. This methodology allows a move away from the traditional three component tissue engineering approach of scaffold/biomolecule attachment mediators/cells to a simpler, two component system of only the scaffold plus cells. As the chemical modification can be conducted immediately after scaffold fabrication, this provides longer shelf life and simplifies the process at point of use (tissue culture), facilitating scale-up and potentially reducing cost.

The proposed mechanism for cell attachment is suggested to be through ionic interactions between the quaternary ammonium functional groups on the surface and oppositely charged phosphate groups present in the phospholipid bilayer of the cell membrane (Li et al. 2014; Schweizer 2009). The lack of cell attachment on the negatively charged anionic cellulose films supports the suggestion that ionic interactions between scaffold and phospholipid bilayer is an important factor in cell attachment. On cationic cellulose, cells appeared to be homogenously distributed across the surface with evidence of significant spreading, demonstrating cell viability on the films, while minimal spreading was observed on the unmodified and anionic cellulose films (reflecting attachment data). Furthermore, trends in cell attachment, after exposure to centrifugal force, were the same as that observed in attachment studies: cells bound to cationic cellulose were least affected by centrifugation, whereas minimal cells remained attached on unmodified and anionic cellulose. In the case of anionic cellulose, the presence of FBS was required to retain even 20% cell attachment.

The Young’s modulus (*E*) defines the elongation stiffness of an elastic material and is the ratio of stress to strain. In tissue engineering it is important that the scaffold has a similar *E* to the surrounding tissue so that it can cope with mechanical wear and also to guide stem cell differentiation (Engler et al. 2006). The value of *E* ≈ 2 MPa measured for these cationic cellulose films suggests potential for application in scaffolds for soft tissues or non-weight bearing bone (Zaborowska et al. 2010).

## Conclusion

Cationic bacterial cellulose films, prepared by grafting with GTMAC, showed significantly increased cell attachment and spreading compared to either unmodified, or oxidised (anionic), bacterial cellulose films. An increase of 70% cell attachment occurred even in the absence of any surface-presented proteins. The modification did not degrade the mechanical properties of the films and only a minimal degree of modification and processing was required to improve cell attachment, which is beneficial, reducing processing steps at the point of tissue culture and obviating the use of animal derived products such as FBS. This novel application of using cationically surface functionalised cellulose for tissue engineering provides a range of opportunities in the development of new scaffolds. While we have focussed on films, as 2D scaffolds, useful for rapid cell viability screening and, by extension for measuring cell kinetics, proliferation and morphology, the methodology would be readily applied to 3D scaffolds and will enhance the application of new technology for forming cellulose structures, e.g. by advanced 3D printing techniques.

## Electronic supplementary material

Below is the link to the electronic supplementary material.

Supplementary material 1 (DOCX 1154 kb)
