# Supplementary material for: Surface modified cellulose scaffolds for tissue engineering
Source: Cellulose (Lond). 2016 Nov 9;24(1):253–67. doi: 10.1007/s10570-016-1111-y (PMC7175690; doi:10.1007/s10570-016-1111-y)
Supplement: Supplementary file 1 — Supplementary material 1 (DOCX 1154 kb) [file 10570_2016_1111_MOESM1_ESM.docx]

Surface Modified Cellulose Scaffolds for Tissue Engineering – Supplementary Information

James C. Courtenay,^a^ Marcus A. Johns,^a,c^ Fernando Galembeck,^d,†^ Christoph Deneke,^d^ [Evandro M. Lanzoni](http://pubs.acs.org/action/doSearch?ContribStored=Lanzoni%2C+E+M),^d^ [Carlos A. Costa](http://pubs.acs.org/action/doSearch?ContribStored=Costa%2C+C+A),^d^ Janet L. Scott,^a,b*^ Ram I. Sharma^a,c*^

^a^ Centre for Sustainable Chemical Technologies, University of Bath, BA2 7AY, UK

^b^ Department of Chemistry, University of Bath, BA2 7AY, UK

^c^ Department of Chemical Engineering, University of Bath, BA2 7AY, UK

^d^ [National Nanotechnology Laboratory, Centre for National Research in Energy and Materials](https://www.google.co.uk/search?q=Laborat%C3%B3rio+Nacional+de+Nanotecnologia,+Centro+nacional+de+Pesquisa+em+Energia+e+Materiais&spell=1&sa=X&ved=0ahUKEwifxPSyuKzJAhWB0BoKHWcZBfgQBQgbKAA), Campinas, São Paulo, Brazil

^†^ Present address: Department of Chemistry, University of Campinas, Campinas, Brazil

^*^ Corresponding authors:

Dr Janet L Scott, Department of Chemistry, Claverton Down, Bath, BA2 7AY, United Kingdom, j.l.scott@bath.ac.uk

Dr Ram Sharma, Department of Chemical Engineering, Claverton Down, Bath, BA2 7AY, United Kingdom, r.sharma@bath.ac.uk

Characterisation of modified cellulose:

***Fig. 1:*** *FTIR spectra for unmodified, cationic (DS = 3.0 ±0.0 %) and anionic (DO = 7.6± 1.0 %) cellulose powders were obtained on a Perkin Elmer Spectrum 100 with a universal ATR sampling accessory; 10 scans were acquired in the range 4000 – 600 cm^-1^. FTIR: prominent bands at 1440 cm^-3^ and 1483 cm^-3^ were attributed to the CH_2_ bending mode and methyl groups of the cationic cellulose substituents in accordance with data published by (Zaman et al). The peak at 1754 cm^-1^ on the anionic cellulose spectrum is attributed to the carboxylic acid C=O stretch.*

**200 100 0 [ppm]**


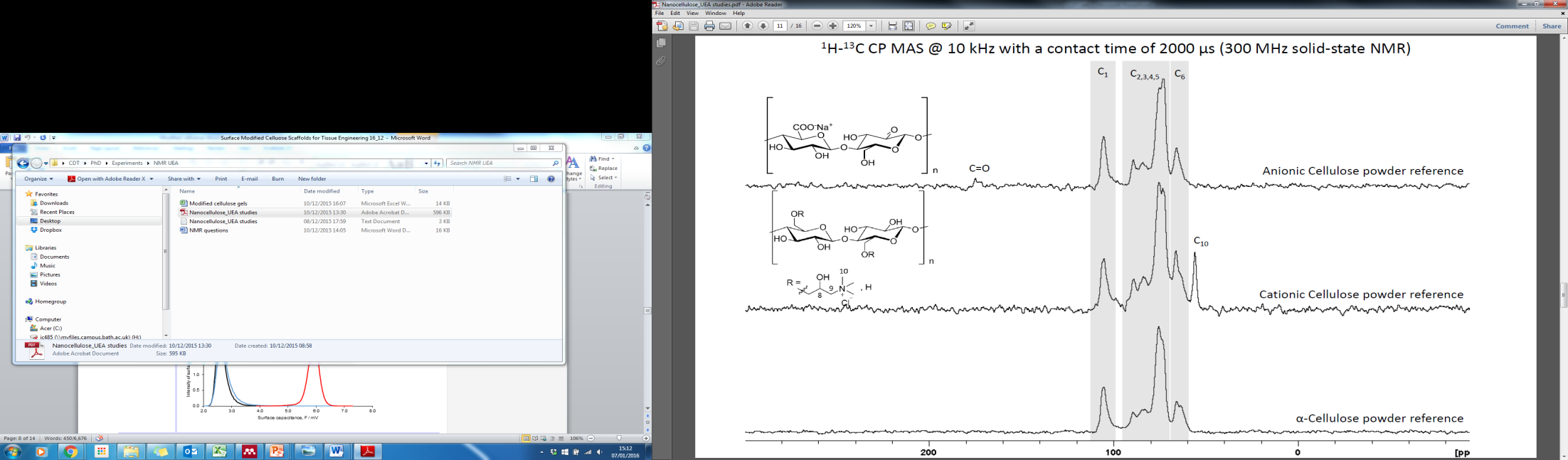


***Fig. 2:*** *^1^H-^13^C CP/MAS NMR was performed on unmodified, cationic (DS = 3.0 ±0.0 %) and anionic (DO = 7.6± 1.0 %) cellulose powders (freeze dried). Spectra were acquired at 25 ^o^C, an MAS rate of 10 kHz and a contact time of 2000 µs. ^13^C solid-state NMR: signals between 66 ppm and 105 ppm referred to the anhydroglucose, while a signal at 175 ppm appeared upon oxidation, due to the carboxylic acid group* *(Saito et al. 2005), and a signal at 56 ppm due to the methyl groups on the quaternary ammonium was detected in the cationic cellulose sample (Chaker et al. 2015).*

| a) | b) |
| --- | --- |
|  |  |

***Fig. 3:*** *a) Conductivity curve for cationic cellulose film in DI H_2_O titrated with ca ~ 1mM AgNO_3_ at 0.50 mL intervals. b) Conductivity curve following an acid/base titration for anionic cellulose film titrated with 0.01 mM NaOH at 0.5 mL intervals (bottom)*

**References:**

Chaker, Achraf, and Sami Boufi. 2015. “Cationic Nanofibrillar Cellulose with High Antibacterial Properties.” *Carbohydrate Polymers* 131: 224–32. doi:10.1016/j.carbpol.2015.06.003.

Saito, T., I. Shibata, A. Isogai, N. Suguri, and N. Sumikawa. 2005. “Distribution of Carboxylate Groups Introduced into Cotton Linters by the TEMPO-Mediated Oxidation.” *Carbohydrate Polymers* 61 (4): 414–19. doi:10.1016/j.carbpol.2005.05.014.

Zaman, Masuduz, Huining Xiao, Felipe Chibante, and Yonghao Ni. 2012. “Synthesis and Characterization of Cationically Modified Nanocrystalline Cellulose.” *Carbohydrate Polymers* 89 (1). Elsevier Ltd.: 163–70. doi:10.1016/j.carbpol.2012.02.066.
